# Use of programmed cell death protein 1 (PD-1) inhibitor therapy in HIV-infected patients with advanced cancer: a single-center study from China

**DOI:** 10.1186/s13027-023-00512-z

**Published:** 2023-05-30

**Authors:** Luling Wu, Jie Su, Junyang Yang, Ling Gu, Renfang Zhang, Li Liu, Hongzhou Lu, Jun Chen

**Affiliations:** 1 Department of Infectious Diseases and Immunology, Shanghai Public Health Clinical Center, Fudan University, Shanghai, China; 2 Institute of Antibiotics, Huashan Hospital, Fudan University, Shanghai, China; 3 State Key Laboratory of Medical Genomics, Shanghai Institute of Hematology, National Research Center for Translational Medicine, Rui-Jin Hospital, Shanghai Jiao Tong University School of Medicine and School of Life Sciences and Biotechnology, Shanghai Jiao Tong University, Shanghai, China; 4 Department of Infectious Diseases and Nursing Research Institution, National Clinical Research Center for Infectious Diseases, The Third People’s Hospital of Shenzhen, Shenzhen, China

**Keywords:** HIV, Cancer, PD-1, ICIs, Safety and outcomes

## Abstract

**Background:**

Anti-PD-1 antibodies have been approved for treating several cancer. However, data regarding the safety and efficacy of these agents in HIV-infected patients with cancer is lacking, because these patients are frequently omitted from clinical trials.

**Objectives:**

The primary aim of our research is to assess the safety, activity, and long-term outcomes of PD-1 inhibitors in the treatment of HIV-infected patients with advanced cancer.

**Method:**

We retrospectively analyzed data from HIV-infected patients with advanced cancers who were treated with PD-1 inhibitors at Shanghai Public Health Clinical Center, Shanghai, China.

**Results:**

Fifteen HIV-infected patients (all are men; asian; median age, 44) with cancer who were treated with chemotherapy and/or combined the other oncology treatments [along with combined antiretroviral therapy (cART)] prior to Sintilimab (12 out of 15) or Nivolumab (1 out of 11) or Camrelizumab (2 out of 11) injection were identified. Eight patients responded to treatment (disease control rate 53.3%), with 1 got partial response (PR) and 7 were stable. Most treatment-emergent adverse events (TEAEs) were grade 1 or 2 including anemia, leukopenia, hyperglycemia, granulocytopenia, and thrombocytopenia. Eight patients (53.3%) experienced treatment-related AEs (TRAEs) with grades 3/4including myelosuppression, infection, and neurological disorders. CD4^+^ T cell count and plasma HIV RNA remained stable throughout the treatment.

**Conclusions:**

When used in HIV-infected patients with advanced malignancies, PD-1 inhibitors tend to have favorable efficacy, manageable side effects, and no deteriorated impacts on plasma HIV-RNA and CD4^+^ T cell count.

## Introduction

HIV infection is related to an increased risk of a range of so-called AIDS-defining cancer including Kaposi’s sarcoma, non-Hodgkin’s lymphoma and cervical cancer [1]. People living with HIV (PLWH) now have a higher survival rate, and the age distribution of the HIV population has altered dramatically as a result of the introduction of cART, with more than one in five PLWH anticipated to be older than 65 by 2030 [2]. Because of this increased longevity, the prevalence of cancer has increased and now includes age-related cancer unrelated to infection. It was reported that PLWH had an increased incidence of non-AIDS-defining malignancies (NADMs), including Hodgkin’s lymphoma and primary cancer of the lung, liver, breast, colon, and prostate [3–7].

T-cell inhibitory receptors are activated on immune cells after T cell receptor (TCR) involvement in balancing chronic antigenic stimulation, and immunological checkpoint inhibitors (ICIs) include PD-1, lymphocyte activation gene 3 (LAG-3), cytotoxic T lymphocyte antigen 4 (CTLA-4) and T-cell immunoreceptor with immunoglobulin and ITIM domain (TIGIT) [8]. ICIs contribute to immune control escape in malignancies and encourage anti-cancer T-cell exhaustion [9]. Anti-PD-1 or anti-PD-L1 antibodies have been authorized for a number of malignancies, such as Hodgkin lymphoma, renal cell carcinoma, and lung cancer, and are currently being studied for almost all forms of cancer. This idea has significantly changed cancer management worldwide [10].

Despite the promising outcomes with anti-PD-1 or anti-PD-L1 antibodies in a variety of cancer, there is a dearth of information on the safety and efficacy of using these drugs in PLWH because these patients are frequently omitted from clinical trials. The inclusion of PLWH in cancer clinical trials has been criticized for a number of reasons, including the potentially reduced effectiveness of ICIs in immunosuppressed people, the potential aggravation of immune reconstitution inflammatory syndrome (IRIS) in PLWH who have started recently cART, the effects of PD-1 inhibitors in the context of perturbations in HIV-related T-cell repertoires, and unknown consequences on other HIV-related malignancies or opportunistic infections [11]. The percentage of PD1^+^ CD4^+^ T and PD1^+^ CD8^+^ T cells in the blood, which play separate roles in HIV persistence during cART, have been found to be significantly correlated in numerous studies [12–14]. Increased PD-1 activity in HIV-positive CD8^+^ T cells and its levels have also been linked to the development of the condition and viremia [15].

To this end, we conducted a retrospective study to demonstrate in more detail of the safety, activity, and long-term outcomes of PD-1 inhibitors in PLWH.

## Methods

### Study population

In this retrospective cohort study, all PLWH treated with PD-1 inhibitors for any cancer at Shanghai Public Health Clinical Center, Shanghai, China from September 2019 to May 2022 were enrolled, regardless of complicating disease, CD4^+^ T cell count, initiating cART or not. Diagnoses of complicating disease were based on H&E staining of tissue and/or cytopathology supported by immunohistochemistry, or microorganism culture, or DNA detection by PCR.

### Treatment

Patients were treated with PD-1 inhibitors (e.g., Sintilimab, Nivolumab and Camrelizumab) according to the standard guidelines and drug instructions. PD-1 inhibitors were used every 3 weeks. Patients were followed up and assessed clinical responses regularly. To evaluate the clinical response to therapy, we used the response evaluation criteria in solid tumors (RECIST). Patients were defined as treatment responders if they met complete remission (CR), PR, or stable disease (SD) according to RECIST criteria [16]. The frequency of TEAEs and laboratory abnormalities graded in accordance with the Common Terminology Criteria for Adverse Events (CTCAE v.4.0) published by the National Cancer Institute served as safety data. Patients were followed every 3-week visit about the appearance of TEAEs associated with the treatment of PD-1 inhibitors. Data were collected before and after PD-1 inhibitors treatment based on medical record review. We gathered data on the patient's demographics, cancer types, drugs administered, response to treatment, and side effects. Additionally, we also investigated trends in CD4^+^ T cell count, plasma HIV viral load and the type of cART therapy. Every 24 weeks, CD4^+^ T cell count and plasma HIV viral load are evaluated as part of HIV patient follow-up. This research was approved by the Ethic Committee of Shanghai Public Health Clinical Center (approval number 2021-S051-01). Due to its retrospective natural, written consent was waived.

### Statistical considerations

The treatment response and immunological reconstitution were evaluated using descriptive statistics. Overall survival (OS) was calculated from the time of initiation of anti-PD-1 antibody therapy until the date of progression, death, or censoring, as applicable, and evaluated using the Kaplan–Meier approach.

## Results

### Patient characteristics

A total of 15 patients were included, all of whom were male. The median (range) age was 44 (29–69) years, all participants were Chinese (Table 1). Seven patients (46.7%) had non-Hodgkin lymphoma (NHL, n = 7). Eight (53.3%) were identified as non-AIDS-defining cancers, including Hodgkin lymphoma (HL, n = 2), lung carcinoma (n = 5) and nasopharyngeal carcinoma (n = 1). Most of the subjects were at late stage of cancer. There was one (6%) participant diagnosed with hypertension. One patient had concomitant hepatitis E and chronic hepatitis B. Thirteen of 15 patients had a median CD4^+^ T cell count of 156 cells/μL (range, 55–375 cells/μL), and 2 patients did not receive a CD4^+^ T cell count test. Three patients had an undetectable baseline plasma HIV viral load and 5 had low-level HIV viremia (less than 200 copies/mL) (Table 2). Six (40%) participants did not report plasma HIV viral load. Six (40.0%) patients had an Eastern Cooperative Oncology Group performance status (ECOG PS) of 0, 8 (53.3%) had an ECOG PS of 1, and 1 (6.7%) had an ECOG PS of 3.

**Table 1:** Clinical characteristics, response, and toxicity profile of patients

Patient	Age (years)	Sex	Cancer and stage	Prior PD-1 inhibitors therapy	ECOG /PS	Best response	Toxicity (grade)	Other treatments combine with PD-1 inhibitors	PD-1 inhibitors treatments and cycles	Time since treatment initiation (months)	Alive or dead
1	57	M	LCLC IV	Chemotherapy + targeted cancer therapy	1	SD	Pneumonitis (4) Leukopenia (2) Hemiparesthesia (2) Granulocytopenia (1) Anemia (1) Thrombocytopenia (1) Hyperglycemia (1) Elevated transaminase (1) Convulsions Dizzy	None	Sintilimab (10 cycles)	7.2	D (Pneumonitis)
2	46	M	DLBCL II	Chemotherapy + targeted cancer therapy	0	PD	Anemia (2) Thrombocytopenia (2) Leukopenia (1) Hyperglycemia (1)	Chemotherapy + targeted cancer therapy	Sintilimab (6 cycles and ongoing)	5.1	Alive
3	29	M	BL IV	Chemotherapy + surgery	3	SD	Leukopenia (4) Granulocytopenia (4) Thrombocytopenia (3) Dyskinesia(3) Septicemia(3) Anemia (2) Pneumonitis (2) Rash (2) Hyperglycemia (1)	Chemotherapy	Sintilimab (34 cycles and ongoing)	27.8	Alive
4	62	M	SCLC IV	Chemotherapy	0	SD	Anemia (1) Mouth ulcers (1)	Chemotherapy	Sintilimab (2 cycles and refuse treatment)	18.3	Alive
5	69	M	SCLC IV	Chemotherapy	1	PD	Anemia (2) Nausea (2) Vomit (2) Hypoglycemia (1) Hypokalemia (1) Hemiparesthesia (1)	Chemotherapy	Sintilimab (3 cycles and refuse treatment)	NA	NA
6	38	M	BL IV	Chemotherapy + targeted cancer therapy + iNKT cell therapy	0	SD	Septicemia(4) Anemia (3) Hyperglycemia (1) Diarrhea (1)	Chemotherapy + targeted cancer therapy	Sintilimab (2 cycles)	1.9	D (Septicemia)
7	50	M	HL IV	Chemotherapy	0	SD	Hyperglycemia (1)	None	Sintilimab (31 cycles and ongoing)	26.1	Alive
8	34	M	NHL IV	Chemotherapy + targeted cancer therapy	0	PD	Anemia (3) Septicemia(3) Leukopenia (2) Granulocytopenia (1) Thrombocytopenia (1) Hyperglycemia (1)	None	Sintilimab (4 cycles and ongoing)	3.8	Alive
9	39	M	DLBCL IV	Chemotherapy + targeted cancer therapy + radiotherapy	1	SD	Septicemia(4) Anemia (3) Nausea (2) Hyperglycemia (1) Fatigue (1) Hypokalemia (1)	Chemotherapy	Sintilimab (3 cycles)	3.7	D (Septicemia)
10	32	M	HL II	Chemotherapy	0	NA	Leukopenia (1) Granulocytopenia (1)	None	Nivolumab (4 cycles and refuse treatment)	39.9	Alive
11	43	M	DLBCL II	Chemotherapy + targeted cancer therapy	1	PD	Leukopenia (4) Granulocytopenia (3) Anemia (3) Thrombocytopenia (2) Hyperglycemia (2) Soft tissue infection (2) Rash (2) Hematochezia (1)	Chemotherapy + targeted cancer therapy	Sintilimab (2 cycles and ongoing)	1.7	Alive
12	44	M	DLBCL IV	Chemotherapy + targeted cancer therapy + radiotherapy	1	NA*	Anemia (4) Septicemia(4) urinary tract infection (1)	Targeted cancer therapy	Sintilimab (1 cycles)	1.2	D (Septicemia)
13	40	M	NPC II	Chemotherapy + radiotherapy	1	SD	Anemia (2) Rash (2) Hearing loss (2) Leukopenia (1) Elevated transaminase (1) Fatigue (1)	Chemotherapy	Sintilimab (9 cycles and ongoing)	6.3	Alive
14	56	M	SCLC IV	Chemotherapy	1	NA*	Weight loss (2) Fatigue (1) Pneumonitis (1)	Chemotherapy	Camrelizumab (1 cycles and ongoing)	1.7	Alive
15	64	M	SCLC IV	Chemotherapy	1	PR	Anemia (4) Septicemia(4) Granulocytopenia (3) Dyskinesia (3) Leukopenia (2) Elevated transaminase (2) Weight loss (2) Leukopenia (1)	Chemotherapy	Camrelizumab (7 cycles)	6.2	D (Septicemia)

*M* cisgender male; *HL* Hodgkin Lymphoma; *NHL* Non-Hodgkin Lymphoma; *SCLC* Squamous Cell Lung Carcinoma; *LCLC* Large Cell Lung Carcinoma; *DLBCL* Diffuse Large B Cell Lymphoma; *BL* Burkitt’s Lymphoma; *NPC* Nasopharyngeal Carcinoma; *ECOG* Eastern Cooperative Oncology Group performance score; *PS*, Performance Status; *NA*, Not Available

^*^Died or follow-up before response could be assessed; SD, Stable Disease; PR, Partial Response; PD, Progressive Disease

**Table 2:** HIV-related markers while on immune checkpoint inhibitor therapy

Patient	cART regimen	plasma HIV viral load (copies/mL) at baseline	plasma HIV viral load (copies/mL) at 24 weeks	CD4^+^ T cell count (cells/*μ*L) at baseline	CD4^+^ T cell count(cells/*μ*L) at 24 weeks
1	TDF + LAM + EFV	< 40	NA	171	159
2	TDF + LAM + DTG	Not detected	Not detected	156	137
3	FTC + TAF + RAL	< 40	Not detected	120	177
4	TDF + LAM + EFV	Not detected	NA	375	NA
5	TDF + LAM + EFV	NA	NA	NA	NA
6	TDF + LAM + DTG	159	NA*	317	NA*
7	TDF + LAM + EFV	< 40	Not detected	208	337
8	TDF + DTG + FTC	198	43.8	60	NA
9	TDF + LAM + DTG	NA	NA*	156	NA*
10	TDF + LAM + EFV	NA	Not detected	NA	824
11	TDF + LAM + DTG	2.18E + 5	NA*	120	NA*
12	TDF + LAM + EFV	NA	NA*	55	NA*
13	BIC + FTC + TAF	NA	Not detected	340	288
14	TDF + LAM + EFV	NA	NA*	155	NA*
15	TDF + LAM + EFV	Not detected	Not detected	121	206

*TDF* Tenofovir Disoproxil Fumarate; *LAM* Lamivudine; *EFV* Efavirenz; *DTG* Dolutegravir; *BIC* Bictegravir; *FTC* Emtricitabine; *TAF* Tenofovir Alafenamide Fumarate; *RAL* Raltegravir; *NA* Not Available

^*^Died or follow-up before response could be assessed

### Treatment

Before anti-PD-1 antibody therapy, 14 patients (93.0%) received chemotherapy, while some subjects also received targeted treatment, surgery, radiotherapy or cell therapy. Eleven patients (73.0%) were treated with PD-1 inhibitors in combination with chemotherapy or targeted cancer therapy. Overall, 12 (80.0%) patients received Sintilimab, 1 (6.7%) patient received Nivolumab, and 2 (13.3%) patients received Camrelizumab at the time of censor. The median cycle was 4 (range, 1–34). They were followed up for 8.2 months (range, 1.2–39.9), by the day of database lock (May 30, 2022). Seven patients continue to be given anti-PD-1 antibody therapy to date.

### Treatment response and Overall survival (OS)

**Fig. 1:**
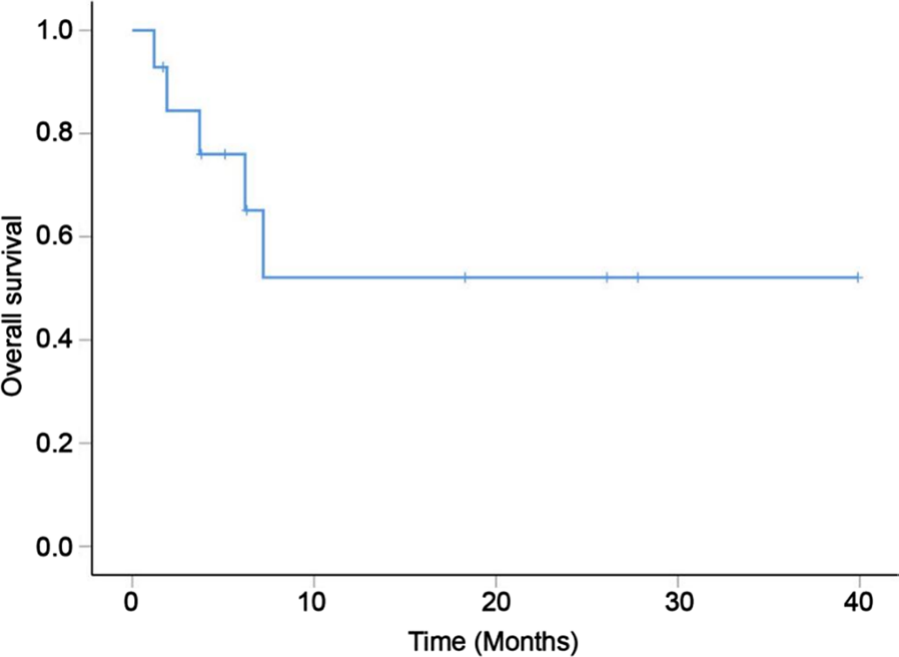
OS of all treated patients

Eight patients responded to treatment, with 1 received PR and 7 got SD, leading to disease control rate (DCR) of 53.3%. The time range of reaching stable conditions is 1–7 cycles. The efficacy of anti-PD-1 antibody therapy was evaluated as progressive disease (PD) in 3 patients with NHL and 1 patient with lung cancer. The efficacy of 2 patients has not evaluated due to the course of treatment was not full. One patient lost to follow-up, unable to assess outcome. When we analyzed data, 9 patients were still alive (4 patients had responded to therapy) and 7 of them were still taking their medication. Five (33.3%) participants had died at the time of analysis due to serious infection. The estimated 1-year OS rate was 66.7% (Fig. 1).

### Safety outcomes

TEAEs that were possibly attributed to anti-PD-1 antibody therapy occurred in all participants (Table 1). Most TEAEs were grade 1 or 2 including anemia (n = 12), leukopenia (n = 9), hyperglycemia (n = 8), granulocytopenia (n = 6), thrombocytopenia (n = 5). TRAEs adverse reactions including myelosuppression, infection, and neurological disorders occurred in 8 (53.3%) of patients. Symptomatic treatment was given for both myelosuppression and infections, all of these participants died due to a serious infection.

### CD4 and HIV monitoring

CD4^+^ T cell count and plasma HIV RNA were monitored during the research (Table 2). The median CD4^+^ T cell count during 24 weeks was 206 cells/μL (the range was 137–824 cells/μL). Two SD patients and one PR experienced increases in CD4^+^ T cell counts within 24 weeks. In 6 individuals for whom data were available, plasma HIV RNA remained below the detection threshold, while plasma HIV RNA continued to decline in patient 11 with PD.

## Discussion

The primary reason for death in PLWH has become cancer [17, 18]. A good response to ICIs therapy in some virus-associated lymphomas has been linked to the increased expression of PD-1 and its ligand PD-L1 in virus-associated cancers [19, 20]. Furthermore, virus-driven cancers, such as Kaposi's, NHLs and cervical cancer, may also increase T cell exhaust due to additional chronic viral stimulation increasing the rationale for using ICIs for these cancer [21]. ICIs have significant promise to treat a wide variety of cancers in PLWH. Given the immune-cell sparing nature, ICIs salvage therapy may exert a beneficial effect on patients' ongoing immunosuppressive chemotherapy. A recent retrospective analysis revealed that Pembrolizumab had a 50% response rate in HIV-associated NHL, suggesting that these drugs should be researched further in HIV-associated NHL [22].To show evidence for use in such a patient population, a few previous data have been completed to evaluate the safety and effectiveness of PLWH [23–26].

Our study is the first paper describing the use of PD-1 inhibitors in Chinese patients with HIV-combined malignancies. We reported 15 PLWH with advanced malignancies who were injected with PD-1 inhibitors. Among 15 patients evaluable for response, the DCR was 53.3%, similar to previous studies [25, 26]. The estimated 1-year OS rate was 66.7%, which also resembled that reported by Kathryn Lurain et al. [22]. These data were significantly higher than that in cancer patients among PLWH without PD-1 inhibitors [27, 28].

The responses were noticed and TRAEs were favorable among most patients. Some of the patients experienced grade 3–4 adverse reactions, including myelosuppression, infection, and neurological. TRAEs occurred in 8 (53.3%) patients, significantly higher than the 20% reported by Gonzalez-Cao M et al. [25]. This study is real-world research, which is closer to clinical reality and more representative. The reason for this disparity may be a result of the fact that patients included in the study were all patients with advanced cancer that had failed after multiple anti-cancer treatments. Ethnicity and the type of pathology are different from that previously reported. The majority of patients (87.5%) who experienced myelosuppression received concomitant chemotherapy or targeted therapy. Thus myelosuppression cannot be excluded in association with oncologic combination therapy. Patients who showed neurological adverse effects were combined with metastatic lesions in the brain. Serious AEs were likely to be generally attributed to complications of progressive cancer in this study. Some patients develop severe infections. However, grade 3 or 4 infections were relatively uncommon in the past PD-1 inhibitors studies (< 1% of patients) [29–32].

The role of ICIs in the management of chronic liver viral infections is likewise of immense clinical interest. Twenty participants with chronic HBV infection who were virally suppressed participated in recent open-label research of Nivolumab with and without the HBV vaccine, and the results demonstrated that Nivolumab was both secure and well-tolerated [33]. In the current study, one patient had concomitant hepatitis E and chronic hepatitis B. The cART was TDF, LAM combined with EFV, and none had a reactivation of HEV or HBV during treatment.

During HIV infection, immune checkpoint proteins have been extensively researched, originally in relation to the virus's natural history and T cell function, but more recently in relation to HIV infection sequelae. T cell exhaustion is a characteristic of many chronic viral infections, including HIV. The expression of multiple immune checkpoint proteins on CD4^+^ and CD8^+^ T cells is upregulated in untreated HIV infection, including PD-1, CTLA-4, LAG-3 and TIM-3 [21, 34, 35]. Many observational studies have shown a strong correlation between clinical outcomes and the expression of PD-1 on CD4^+^ or CD8^+^ T cells. Increased expression of PD-1 was linked to an accelerated decline in the number of CD4^+^ T cells after acute infection and untreated chronic infection in the absence of ART [34, 36]. ICIs may also be promising candidates for “shock and kill” treatment strategies because they can reactivate the HIV-1 reservoir [37, 38], while simultaneously strengthening antiviral immune responses [34, 39], consequently reducing the HIV-1 reservoir size. Consistent with the study by Uldrick et al. [40], our results confirm that PD-1 inhibitor therapy is likewise safe in terms of sustained control of HIV infection. All patients with available data had stable CD4^+^ T cell counts. The present investigation showed that CD4^+^T cell counts do not seem to be adversely affected by PD-1 inhibitors. In participants for whom data were available, plasma HIV viral load remained suppressed below the threshold for detection. No patients required a change of cART. We didn’t observe any autoimmune disorder activation or episodes. Due to the fact that this trial only included peripheral blood samples from a small number of patients who underwent suppressive cART that completely controlled viral replication, any positive effects of PD-1 inhibitor therapy on plasma HIV viral load may have been obscured. Therefore, comprehensive analysis of HIV reservoir, including cell-associated HIV RNA and HIV DNA should be performed. Meanwhile, given interindividual heterogeneity, these restrictions must be properly taken into account, especially for cancer participants. The potential role of ICIs in treatment approaches should be further recognized by ongoing clinical trials of ICIs alone or in combination with latency reversal medicines for cART in HIV-1-infected individuals [25, 41].

All patients in this study received standard oncology treatment before anti-PD-1antibody therapy, which potentially increased the positive effects of PD-1 inhibitors. To date, cancer regression or stabilization was noted in 3 patients with lung cancer, 3 patients with NHL, 1 patient with HL, and 1 patient with NPC. Clinical benefits were observed in lung cancer, HL, and NHL. It is consistent with the findings of the most recent Phase 1 study to assess the security of Pembrolizumab in PLWH with advanced cancers [38]. Despite a small number of patients, it is impossible to exclude the fact that cART which reconstituted the immune system of PLWH, may also improve their anticancer activity.

Our study aims to strengthen knowledge regarding the efficacy of ICIs in PLWH with advanced cancer. There is no doubt that HIV and infectious disease professionals should collaborate closely with oncologists in order to promote secure cancer and PLWH treatment options. A multidisciplinary approach is needed in the diagnosis and management of PLWH and cancer, where feasible.

In conclusion, our retrospective study suggests that PD-1 inhibitors treatment may be both effective and safe for PLWH with cancer. This study is limited by the small sample size. Meanwhile, all the patients included were male, as PLWH are more commonly male in China, as well as those with cancer [27, 42]. Larger research is required to validate the promising and favorable anti-cancer activity with PD-1 inhibitors treatment for PLWH. HIV-infected patients with advanced cancer should be included in future cancer clinical trials.

## Data Availability

All data generated or analyzed during this study are included in this article. The other raw datasets used and/or analyzed in this study will be made available upon reasonable request to the corresponding author.
